# Prokaryotic Diversity of Ruminal Content and Its Relationship with Methane Emissions in Cattle from Kazakhstan

**DOI:** 10.3390/life12111911

**Published:** 2022-11-17

**Authors:** Aida Daugaliyeva, Saule Daugaliyeva, Alexander Ashanin, Chiara Beltramo, Latipa Mamyrova, Zinagul Yessembekova, Simone Peletto

**Affiliations:** 1 LLP ‘Kazakh Research Institute for Livestock and Fodder Production’, St. Zhandosova 51, Almaty 050035, Kazakhstan; 2 LLP ‘Scientific Production Center of Microbiology and Virology’, Bogenbay Batyr Str. 105, Almaty 050010, Kazakhstan; 3 Istituto Zooprofilattico Sperimentale del Piemonte, Ligura e Valle d’Aosta, Via Bologna 148, 10154 Turin, Italy

**Keywords:** cattle, methane, rumen, microbiota, 16S metabarcoding, Kazakhstan

## Abstract

In this study, we analyzed the microbial composition of the rumen contents of cattle from Kazakhstan. Specifically, samples of the liquid and solid fractions of the rumen were collected to determine the quantitative and qualitative composition of methanogenic archaea. Cattle were six steers receiving hay-concentrate feeding. Methane emission was determined by repeated measurements for each animal. Rumen samples were then taken from fistulas and analyzed using 16S metabarcoding via Next-Generation Sequencing (NGS). The difference between the rumen fractions was investigated, resulting in differential distribution of the families *Streptococccaceae, Lactobacillaceae, Desulfobulbaceae,* and *Succinivibrionaceae*, which were more abundant in the liquid fraction, while *Thalassospiraceae* showed a higher presence in the solid fraction. These differences can be explained by the fact that fibrolytic bacteria are associated with the solid fraction compared to the liquid. A relationship between methane emission and methanogenic microbiota was also observed. Steers producing more methane showed microbiota richer in methanogens; specifically, most *Mathanobacteriaceae* resided in the liquid fraction and solid fraction of animals 1 and 6, respectively. The same animals carried most of the *Methanobrevibacter* and *Methanosphaera* genera. On the contrary, animals 2, 3, and 5 hosted a lower amount of methanogens, which also agreed with the data on methane emissions. In conclusion, this study demonstrated a relationship between methane emission and the content of methanogenic archaea in different rumen fractions collected from cattle in Kazakhstan. As a result of the studies, it was found that the solid fraction of the rumen contained more genera of methanogens than the liquid fraction of the rumen. These results prove that taking rumen contents through a fistula is more useful than taking it through a probe. The presented data may be of interest to scientists from all over the world engaged in similar research in a comparative aspect.

## 1. Introduction

The rumen contains one of the most complex microbial communities—prokaryotes, eukaryotes, and viruses. Prokaryotes include bacteria, primarily anaerobic bacteria, and archaea, including methanogens [1]. They have a role in digesting fibrous feed and providing nutrients to the animal host. There is an environmental cost, however, in which ruminants, via their ruminal microbiota, produce substantial amounts of greenhouse gas (GHG), i.e., methane [2,3,4]. The rumen is an anaerobic and methanogenic fermentation chamber that contains microorganisms that can utilize and increase the productivity of cellulolytic feeds (e.g., straw, hay, silage, and grass). Understanding the rumen microbiota and their connection to the ruminant itself is important for producing quality products, increasing profitability, and reducing environmental impacts [5]. Moreover, methane cannot be absorbed by the host, as it represents excess energy [6] and is therefore emitted to the environment. The methane production in the rumen is 2–12% of the total energy consumed, which could be used instead to increase the body weight of animals [7].

There are three intersecting microenvironments found in the rumen, which contain bacteria, archaea, protozoa, and fungi: the liquid phase, making up 25% of the microbial mass; the solid phase, making up 70% of the microbial mass; and the rumen epithelial cells and protozoa, containing 5% of the microbial mass [8]. In terms of cell numbers, bacteria are the most abundant, representing over 95% of microorganisms within the rumen ecosystem [9], and were first described using classical microbiology methods [10]. When cattle are fed a high amount of grains, the relative abundances of the members of the phyla *Bacteroidetes* and *Fibrobacter* in the microbial communities in the digestive tract decline, allowing *Firmicutes* and other opportunistic phyla, such as Gram-negative *Proteobacteria*, to proliferate faster, resulting in an increase in the proportions of *Firmicutes* and *Proteobacteria* [11].

Rumen archaea are strictly anaerobic and are the only known microorganisms present in the rumen capable of producing methane [12]. Such archaea are referred to as methanogens. Archaea are found in the rumen in the range of 10^6^ to 10^8^ cells per ml, accounting for less than 4% of the microbial community [13]. Wallace R.J. et al. [14] identified the *Megasphaera* genus as being significantly enriched in the rumen of low- methane-emitting cows.

The fiber-adherent microbiota is characterized by a higher proportion of taxa associated with fiber degradation, such as *Ruminococcaceae* and *Fibrobacter*, whereas the liquid phase is characterized by enrichment in members of the *Prevotellaceae* family, capable of utilizing a broad range of soluble substrates [15,16]. Studies by Brulc et al. [9] were performed on the rumen of three steers, revealing that one steer had a remarkably different microbiota composition compared with the other two steers analyzed, despite similar housing and dietary conditions and without any obvious differences in the animals’ physiology. Other authors had similar conclusions [17,18]. This observation is not limited to bacteria, and similar inter variation within the same host species can be seen in the other microbial domains inhabiting the rumen [19,20,21].

This study aimed to characterize the microbiota of liquid and solid fractions of the rumen in cattle from Kazakhstan, as well as investigate the association of identified bacteria and methanogens with methane production.

## 2. Materials and Methods

### 2.1. Animals

The experiment followed the rules for the treatment of animals (order of the Minister of Agriculture of the Republic of Kazakhstan dated 30 December 2014, no. 16-02/701) and was approved by the Bioethics Commission of the Kazakh Research Institute for livestock and Fodder Production. The research was carried out in the experimental farm of the ‘Kurazek’, Institute Zhambyl district, Almaty region. The animals enrolled in the study were 6 steers belonging to different breeds (i.e., Kazakh white-headed, Alatau, Simmental, and Holstein breeds) with a live weight of 350–400 kg and an average daily increase in live weight of 1200–1500 g. They were healthy animals of the same age (15 months old).

### 2.2. Average Daily Rations

The animals were fed alfalfa hay, straw, and concentrates. The average daily nutritional value of the diet was 11.9 EFU. The experiment duration was 52 days; then, sampling was carried out. The chemical analysis of feed was carried out in accordance with state standard 32040-2021. To determine the chemical composition of feed, a modern Infrasac device from FOSS (Denmark) was used.

The average daily ration was developed and calculated based on data on the amount of given feed; their palatability; and the actual consumption of feed, nutrients, and biologically active substances. The average daily ration characterizes the background and type of feeding for the period of experience. The average daily rations of animals’ actual consumed feed for the study period are given in Table 1.

### 2.3. Methane Measurement

**Figure 1:**
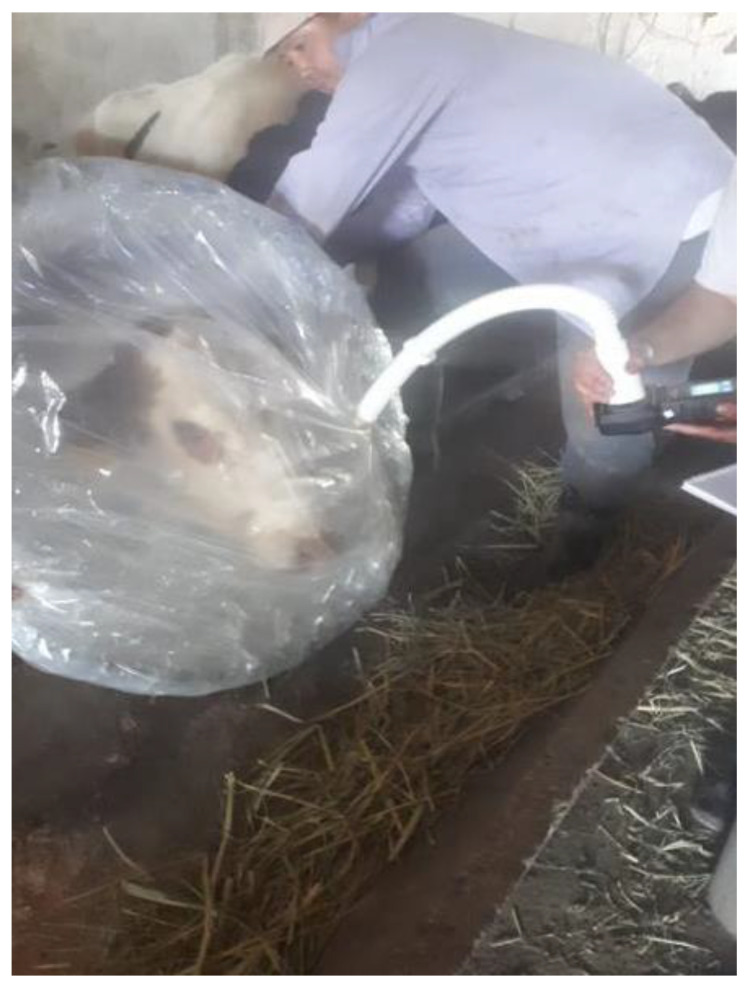
Measurement of methane emission using the gas analyzer ‘Signal-44′.

During the experiment, the methane concentration in the exhaled air was measured for each animal four times a day for three days. Measurements of methane were carried out by the gas analyzer ‘Signal-44′ (TU 4215-002-80703968-07, according to the operating manual of the device). To ensure accurate analysis, the animal’s head was covered with plastic wrap (Figure 1). The operation of the gas analyzer was based on measuring the electrical signal coming from the gas-sensitive sensor (thermal catalytic sensor), which is proportional to the concentration of the measured substance within the measuring range. Methane measurement was carried out based on the Sniffer Method 22 with modifications.

### 2.4. DNA Extraction, PCR Amplification, and 16S Metabarcoding

**Figure 2:**
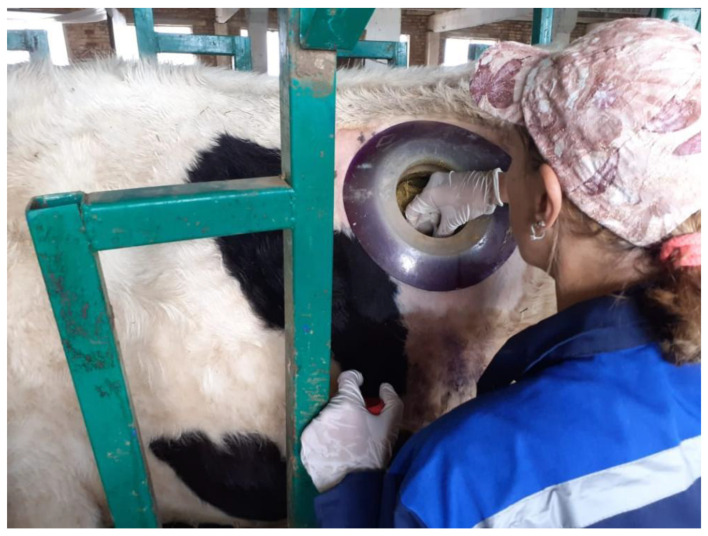
Sampling from the rumen through the cannulated hole.

The rumen contents were mixed manually, and then an aliquot was collected (Figure 2, Table 2). The liquid fraction was separated from the solid content by filtration through sterile cheesecloth. All the samples were transported on dry ice to the LLP ‘Scientific Production Center of Microbiology and Virology’ in Almaty for DNA extraction, amplification, and analysis. Samples from the solid and liquid phases were handled separately.

Two hundred mg of ruminal samples was taken for DNA isolation. DNA from the samples was isolated using the PureLink^TM^ Microbiome DNA Purification Kit (Invitrogen, Carlsbad, CA, USA) according to the manufacturer’s protocol. In addition to the samples, a positive control consisting of a standard microbial community (MOCK, Zymo Research) and a negative extraction control was set up. The DNA concentration was measured by a Qubit^®^ 2.0 fluorimeter using a Qubit™ dsDNA HS Assay Kit (Life Technologies, Eugene, Oregon, USA). The genetic libraries were prepared for sequencing following the 16S Metagenomic Sequencing Library Preparation guide (part no. 15044223 rev. A). As an extraction control, a lysis buffer was used from a DNA isolation kit without any biological material. Molecularly pure water was used to control the libraries and was added to the amplification mixture. The amplification product was not observed in the controls. The libraries pooled with PhiX were sequenced on an Illumina MiSeq device using a 600-cycle MiSeq^®^ Reagent Kit v3 (Illumina Inc., San Diego, CA, USA), following the manufacturer’s recommendations.

### 2.5. Bioinformatics and Data Analysis

The 16S metabarcoding data obtained from the rumen samples were analyzed using the Data QC workflow. The raw fastq data were analyzed with the ‘Data QC and OTU Clustering’ and ‘Estimate Alpha and Beta Diversity’ workflow tools of the Microbial Genomics Module in the CLC Genomic Workbench software (Qiagen). The paired-end reads were joined and trimmed for low quality score (Qscore < 0.05), nucleotide ambiguity (max of 2 nucleotides allowed), adapter sequences, and length. Duplicate sequences were merged and aligned against the SILVA database (v. 132) at 97% identity threshold. Chimeric reads were removed, and taxonomy was assigned, creating an OTU table. The profiles of the negative control and the mock communities were analyzed to check for correct procedures and cross-contamination, then removed. Rarefaction curves did not reach a plateau in all study samples, so they were compared at a sequencing depth of 50,000 reads. The alpha diversity (diversity within the groups) was estimated using the total number, Chao-1 bias-corrected, Simpson’s index, and Shannon entropy. The Bray–Curtis method, Jaccard, Unweighted UniFrac and Weighted UniFrac, and Principal Coordinates Analysis (PcoA) were used to estimate the beta diversity (diversity between groups). Statistical support was calculated using the Kruskal–Wallis test for alpha diversity and the PERMANOVA test for beta diversity (*p*-value ≤ 0.05). The OTU table showing the abundance of the genera in the analyzed samples was used to perform a generalized linear model test of differential abundance to compare microbial abundance of solid and liquid fractions. The ‘Differential Abundance Analysis’ tool in CLC Genomic Workbench performs a TMM normalization to make samples comparable, adjusting library sizes. The Wald test was used to determine significance between group pairs. Differential abundance analyses compared genera and families by liquid/solid fraction.

## 3. Results

### 3.1. Dynamics of Live Weight of Animals

Roughage and concentrates in the structure of the diet comprised 57.43% and 42.57%, respectively. Succulent foods were excluded from the diet. The eatability of hay was 86.8%, that of straw was 82.1%, and concentrates were consumed completely. On average, the animals consumed 12.6 kg of dry matter per day. The concentration EFU in 1 kg of dry matter was 0.94 MJ; the crude protein content was 156.36 g. Thus, the rations almost completely provided the animals’ need for basic nutrients, which positively affected their productivity (Table 3).

The data given in Table 3 shows that the average daily gain in live weight averaged 1476.0 ± 115.36 g, and the feed cost per 1 kg of gain was 8.1 ± 0.74 ECU.

### 3.2. Methane Emissions from Animals

The largest amount of methane in the exhaled air was found in animals 1 and 6. The averaged data on methane emission are shown in Table 4. Methane yield, in our case, is the concentration coefficient.

### 3.3. Rumen Microbiota

**Figure 3:**
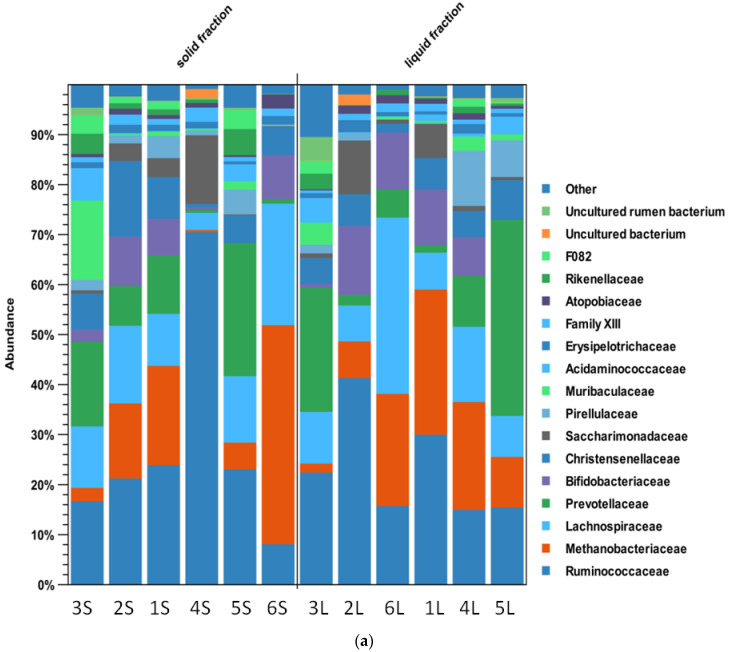

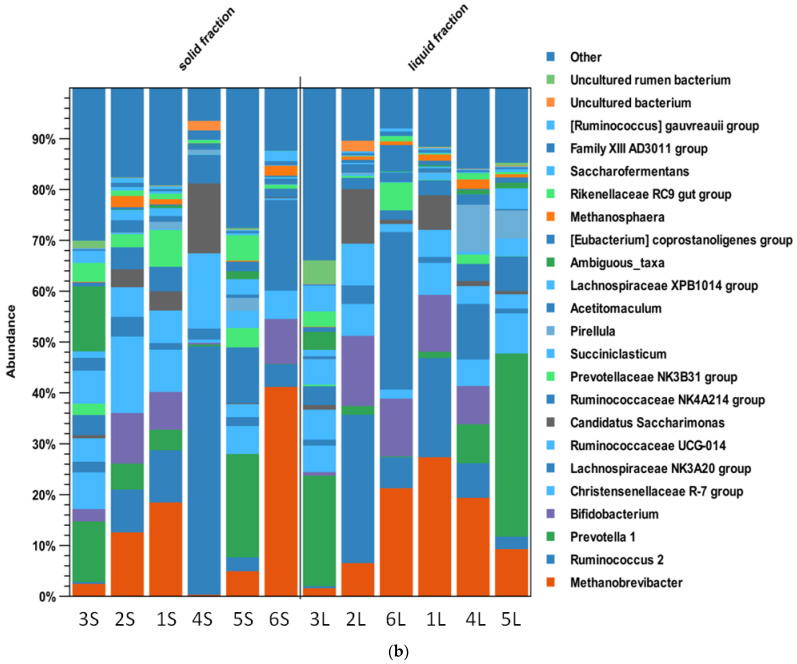
(**a**). Histogram at the family level of the microbiota of each sample, compared by rumen fraction. (**b**). Histogram at the genus level of the microbiota of each sample, compared by rumen fraction.

The high throughput sequencing of the V4 region of the 16S rRNA gene produced 3,963,970 reads; 3,959,335 paired reads were obtained for OTU clustering after quality filtering. The 392,411 unique nonchimeric sequences were assigned to 2,447 OTUs. Data from the mock community analysis matched the expected results, while the negative controls showed a low number of reads assigned to OTUs absent or with negligible abundance in the samples. Histograms comparing fractions (Figure 3a,b) showed differences at the family and genus level, not always significant after statistical analysis (as shown later in the text).

**Figure 4:**
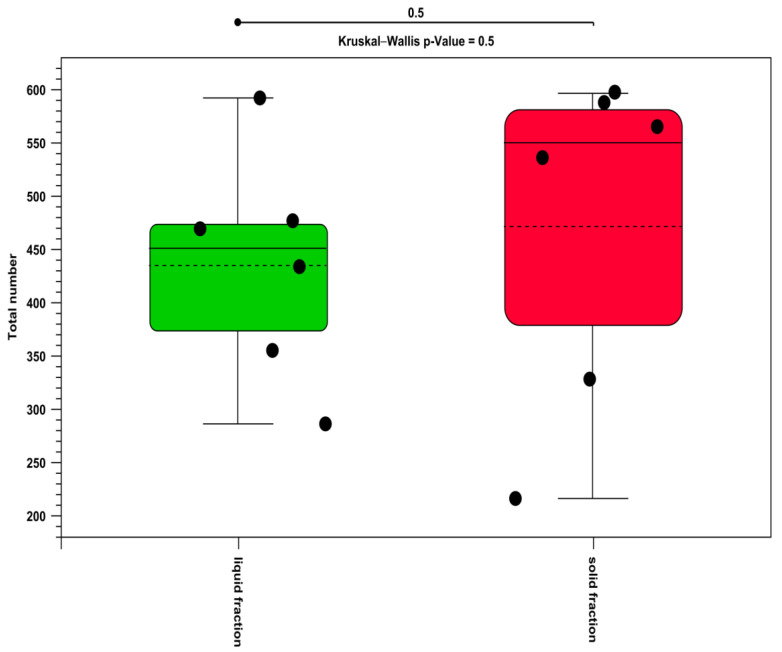
Alpha-diversity evaluated by comparing the fraction (green = liquid fraction; red = solid fraction).

The alpha diversity was insignificant for all the compared samples with regard to fraction comparison. All the *p*-values were >0.05 for each of the different estimations. For example, the alpha-diversity results estimated by the total number comparing ruminal fractions are reported in Figure 4.

**Figure 5:**
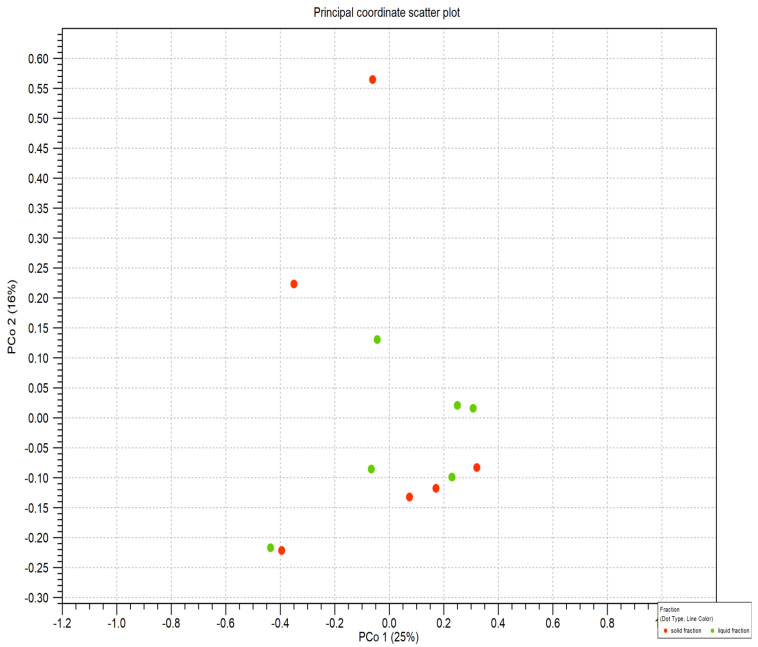
Scatter plot of the Beta diversity comparing rumen fractions (green—liquid fraction; red—solid fraction).

No differences were identified by considering the fraction by beta diversity analysis. As an example, in Figure 5, the results of the Bray–Curtis estimation are shown.

However, the differential abundance analysis showed significant differences for specific taxonomic groups comparing the liquid and solid fractions (Table 5).

At the family level, *Streptococcaceae, Lactobacillaceae, Desulfobulbaceae,* and *Succinivibrionaceae* were more abundant in the liquid fraction, while *Thalassospiraceae* showed a higher presence in the solid fraction. At the genus level, *Ruminobacter, Howardella, Streptococcus, Lactobacillus, Ruminococcaceae* UCG-013, *Eubacterium Сoprostanoligenes*, *Succinivibrionaceae*, *Desulfobulbus,* and *Psychrobacter* showed a higher abundance in the liquid fraction, while *Thalassospira* was more abundant in the solid fraction.

In the liquid fraction, the largest relative number of representatives of the families *Ruminococcaceae* (41%), *Bifidobacteriaceae* (14%), and *Saccharimonadaceae* (11%) was in 2L. The families *Prevotellaceae* (39%) and *Christensenellaceae* (8%) prevailed in another animal (5L). The family *Lachnospiraceae* (35%) was the most abundant in 1L. In 4L, the *Pirellulaceae* family was prevalent (11%). Among methanogens, a large relative number of members of the *Methanobacteriaceae* family (29%) was detected in 1L.

In the solid fraction, the largest relative number of representatives of the families *Ruminococcaceae* (70%) and *Saccharimonadaceae* (14%) was in 4S. In 3S, a large proportion of the families *Muribaculaceae* (16%) and *Acidaminococcaceae* (6%) was observed. The family *Lachnospiraceae* (24%) was also the most represented in 6S. The families *Christensenellaceae* (15%) and *Bifidobacteriaceae* (10%) prevailed in 2S. Additionally, in another animal (5S), the *Prevotellaceae* family prevailed (27%). Among methanogens, a large relative number of members of the family *Methanobacteriaceae* (44%) was found in 6S.

At the genus level, the largest relative number of representatives of the genera *Candidatus Saccharimonas* (41%), *Ruminococcus* 2 (29%), *Bifidobacterium* (14%), and *Ruminococcus* (15%) was in the liquid fraction collected from animal 2. The genus *Prevotella* (36%) prevailed in 5L. The family *Lachnospiraceae* NK3A20 (31%) was the most abundant in 6L. In 4L, the genus *Pirellula* prevailed (9%). Among methanogens, a large relative number of members of the genus *Methanobrevibacter* (27%) was in 1L and the genus *Methanoshaera* (2%) in 4L.

In the solid fraction of the rumen, the largest relative number of representatives of the genera *Ruminococcus* (64%) and *Candidatus Saccharimonas* (14%) was in animal 4S. In another animal (3L), a large number of reads assigned to an unidentified *Muribaculaceae bacterium* (13%) and the genus *Succiniclasticum* (6%) were observed. The family *Lachnospiraceae* (18%) and the genus *Bifidobacterium* (9%) were the most abundant in 6S. The family *Ruminococcaceae* (33%), *Pirellulaceae* (20%), *Christensenellaceae* (15%), *Lachnospiraceae* (15%), and *Bifidobacteriaceae* (10%) prevailed in 2S. Additionally, in 5S, the genus *Prevotella* 1 prevailed (8%). Animal 1 was dominated by the family *Christensenellaceae* R-7 group (8%) and the *Prevotellaceae* NK3B31 group (7%) in both liquid and solid fractions.

Among methanogens, a large relative number of members of the *Methanobrevibacter* genus was found in animals 1 and 6 (41%) and *Methanoshaera* in animals 2 and 6 (2%). The genus *Prevotella1* showed a lower abundance in animal 6 compared with other animals. Animal 1 showed significant differences in *Eubacterium ventriousum* (*Lachnospiraceae* family), which was almost absent in all the other animals.

## 4. Discussion

In this study, the microbiota of liquid and solid fractions of the rumen in cattle from Kazakhstan was characterized, and the association of identified bacteria and methanogens with methane production was determined. 

In previous studies, we determined how the microbiota of cattle affects methane in different regions of Kazakhstan. For this purpose, fecal samples were taken rectally from 37 cattle heads from 4 regions of Kazakhstan (Western, Southern, Northern, and Southeast Kazakhstan). As a result of the studies, it was found that methanogenic bacteria were less present in animals from the Western region grazing in the pasture compared with animals from other regions kept in the stall [22].

Diet is a major factor impacting the microbiota composition of the gastrointestinal tract. As a whole, since the feeding was the same for all animals (i.e., hay-concentrated feeding), the rumen microbiome was dominated by the families *Lachnospiraceae*, *Bifidobacteriaceae*, *Prevotellaceae*, and *Succinivibrionaceae*.

Members of the *Lachnospiraceae* family are Gram-positive, anaerobic, rod-shaped bacteria that predominantly ferment pectin [23]. Li and Guan [24] found that *Lachnospiraceae* are more abundant in the rumen of steers with the lowest average daily gain (ADG) and highest residual feed intake (RFI); however, these observations are not consistent with the findings of Myer et al. [25], who found that *Lachnospiraceae* are more abundant among steers with the highest ADG. Many *Lachnospiraceae* produce butyrate, which is a gut nutrient, and VFAs are produced from carbohydrate digestion [26].

Gagen et al. [27] found that acetogens exist in the *Lachnospiraceae* and *Ruminococcaceae* families. Acetogens can serve as a hydrogen scavenger and can increase if methane production is reduced. The presence of *Lachnospiraceae* and *Ruminococcaceae* indicates a more complete fermentation and an increase in digestible nutrients available to the animal [28]. The genus *Ruminococcus* plays an important role in the decomposition of cellulose [14].

The family *Succinivibrionaceae* (phylum *Proteobacteria*) was abundant in the liquid fraction of the rumen and predominated in grain-feeding animals [29]. The family *Succinivibrionaceae* are propionate producers and promote succinate. The family *Succinivibrionaceae* is known to inhibit methanogenesis [30].

*Prevotella* is usually found in large numbers and has many functions, such as breaking down polysaccharides and proteins. Classical studies showed that metabolic differences are widespread among the population, as they are often found in the rumen with different diets, and have also found that this genus grows freely at a pH of only 5.1 [23]. Delgado et al. [31] found a positive correlation between nutritional efficiency and the genus *Prevotella*. However, other authors note that the number of *Prevotella* in the rumen is greater in steers with the lowest ADG [26]. Previous analysis of the rumen microbiota showed that some OTUs belonging to the genus *Prevotella* were stimulated by the inhibition of methanogenesis. The *Prevotella* species includes propionate production by randomization upon inhibition of methanogenesis [32].

The genera *Lactobacillus* and *Streptococcus* showed a higher abundance in the liquid fraction of the rumen. These genera are recognized producers of lactic acid (lactate) and have higher relative abundance in grain-fed cattle with an acidic rumen environment (acidosis) [29]. Moreover, *Streptococci* produce toxic compounds by fermenting proteins [33] The genus *Eubacterium ventriousum* of the family *Lachnospiraceae* was only found in animal 1 and was nearly absent in all other animals. *Eubacterium* and *Bifidobacterium* are beneficial bacteria due to their ability to synthesize vitamins, aid digestion, stimulate immune function, and suppress pathogenic microbes.

Thus, the solid and liquid fractions of the rumen are different since fibrolytic bacteria are associated with the solid fraction compared with the liquid one. Therefore, for a complete picture of the diversity of the rumen, it is necessary to study both fractions.

To assess any relationship between GHG emission and microbiota composition, the first step was to measure methane emission in the studied animals. The existing methods for measuring methane are numerous, but they also have drawbacks and are unreliable. The need to improve and search for new methods for measuring methane in animals is reported by many authors [34,35,36]. To overcome this issue, multiple measurements were taken for each animal.

Among hydrogenotrophic methanogens, a relatively large number of members of the genus *Methanobrevibacter* (27%), the most common genus of methane producers in the rumen, was found in the liquid fraction of animal 1 and the solid fractions of animals 6 and 1 (41%); the genus *Methanoshaera* was detected in 4L (2%) and 6S (2%). *Methanobrevibacter* spp. is an important methanogen, accounting for 75–78% of methanogenic archaea in the rumen. Interestingly, *Methanosphaera* spp. is a methylotrophic methanogen that produces methane through a primary ethanol-dependent, hydrogen-independent methanogenesis pathway [36]. Family *Methanobacteriaceae* (15%) was observed in sample 2S.

Animal 1L had more abundant *Methanobacteriaceae* in the liquid fraction of the rumen, while animal 6S had more abundant *Methanobacteriaceae* in the solid fraction. The family *Succinivibrionaceae* suppressed methanogenesis and dominated in greater numbers in 3L. The genus *Prevotella* prevailed in sample 5L. The genus *Prevotella1* showed a lower abundance in animal 6, which is consistent with the findings that the genus *Prevotella* inhibits methanogenesis.

Animals 3 and 4 had large numbers of members of the *Succinovibrionaceae* family in their rumen, which alters acetate and hydrogen production, resulting in less methanogenesis and, therefore, reduced methane emissions [14]. The family *Succinovibrionaceae* uses hydrogen to produce succinate, which is then rapidly converted to propionate and competes with the most common hydrogenotrophic methanogenesis.

In this study, we studied how the microbiota collected from the ruminal content of cattle affects methane. The animals involved in the experiment belonged to the Kazakh Research Institute for Livestock and Fodder Production. Due to the technical difficulty and high material costs of fistula installations, only six animals participated in the experiment.

## 5. Conclusions

The study results showed a differential distribution of the rumen microbiota, where *Streptococccaceae*, *Lactobacillaceae*, *Desulfobulbaceae*, and *Succinivibrionaceae* were more abundant in the liquid fraction, while *Thalassospiraceae* was more abundant in the solid fraction. These differences can be explained by the fact that fibrolytic bacteria are associated with the solid fraction compared with the liquid. Moreover, a relationship between methane emission and methanogenic microbiota was demonstrated. Steers producing more methane showed a microbiota richer in methanogens, especially *Methanobrevibacter* and *Methanosphaera* genera. As a result of the studies, it was found that the solid fraction of the rumen contained more genera of methanogens than the liquid fraction. These results prove that taking rumen contents through a fistula is more useful than taking it through a probe. This research is novel to Kazakhstan and adds to the growing global databases that have been developed by other countries under programs such as the global rumen census. The information developed from the project can also help the government to develop a national GHG emission policy.

## Figures and Tables

**Table 1:** Average daily feeding rations of experimental steers’ actual consumed feed (per 1 head).

Name	Quantity
Alfalfa hay, kg	7.81
Straw, kg	1.64
Concentrates, kg	5
The diet contains	
Energy feed units, Mj	11.9
Exchange energy, Mj	119.4
Dry matter, kg	12.6
Crude protein, g	1970.1
Digestible protein, g	1350.8
Split protein, g	1518.7
Non-degradable protein, g	451.4
Crude fiber, g	2701.6
Crude fat, g	271.4
Sugar, g	236.1
Starch, g	2887.1
Calcium, g	140.2
Phosphorus, g	33.7
Carotene, mg	390.9
Iron, mg	2360.8
Zinc, g	360.8
Manganese, g	500.0
Copper, mg	107.3
Cobalt, mg	2.9
Iodine, mg	4.3

**Table 2:** List of the analyzed samples.

Sample Name	ID	Fraction	Animal Breed
1L	SimJ	Liquid	Simmental
1S	SimTv	Solid	Simmental
2L	AlJ	Liquid	Alatau (local breed)
2S	Al T_B_	Solid	Alatau (local breed)
3L	8 BelKJ	Liquid	Kazakh white-headed
3S	7 KBKTv	Solid	Kazakh white-headed
4L	T-1 J	Liquid	Kazakh white-headed
4S	T1-Tv	Solid	Kazakh white-headed
5L	T2-J	Liquid	Alatau (local breed)
5S	T2-Tv	Solid	Alatau (local breed)
6L	GKrJ	Liquid	Holstein-Frisian
6S	GKrTv	Solid	Holstein-Frisian

**Table 3:** Dynamics of live weight of experimental steers.

Indicators	Result
Average live weight of steers when setting up for experiment, kg	394 ± 16.4
Live weight of steers at the end of the experiment, kg	470 ± 24.6
Absolute increase in live weight, kg	76.8 ± 8.2
Average daily live weight gain, g	1476 ± 115.4
Total feed costs, energy feed unit	619 ± 35.7
Feed costs per 1 kg of live weight gain, EFU	8.1 ±0.74

**Table 4:** Concentration of methane in exhaled gases of steers (average for the group, %).

Gas (Volumetric Share, %)	Steers					
	1	2	3	4	5	6
Methane (CH4)	4.02	3.92	3.05	3.63	3.21	4.4
Carbon dioxide (CO_2_)	3.62	3.11	2.92	3.31	3.91	4.54

**Table 5:** Results of differential abundance analysis for liquid vs. solid fractions.

Liquid Fraction vs. Solid Fraction			
Family	Log₂ Fold Change	Fold Change	*p*-Value
*Streptococcaceae*	3.14	8.79	0.005
*Lactobacillaceae*	6.35	81.33	0.010
*Desulfobulbaceae*	2.48	5.57	0.013
*Succinivibrionaceae*	3.64	12.47	0.021
*Thalassospiraceae*	−4.23	−18.72	0.035
Genus	Log₂ Fold Change	Fold Change	*p*-Value
*Ruminobacter*	5.13	34.95	0.002
*Howardella*	3.23	9.41	0.005
*Streptococcus*	3.02	8.12	0.006
*Lactobacillus*	6.71	104.97	0.007
*Ruminococcaceae UCG-013*	2.68	6.40	0.016
*[Eubacterium] coprostanoligenes group*	2.02	4.06	0.026
*Thalassospira*	−4.40	−21.14	0.028
*Succinivibrionaceae UCG-002*	3.31	9.91	0.033
*Desulfobulbus*	2.39	5.22	0.036
*Psychrobacter*	4.79	27.70	0.040

## Data Availability

The data presented in this study are publicly available at the NCBI Sequence Read Archive (Bioproject ID: PRJNA854754).
